# Secreted Herpes Simplex Virus-2 Glycoprotein G Modifies NGF-TrkA Signaling to Attract Free Nerve Endings to the Site of Infection

**DOI:** 10.1371/journal.ppat.1004571

**Published:** 2015-01-22

**Authors:** Jorge Rubén Cabrera, Abel Viejo-Borbolla, Nadia Martinez-Martín, Soledad Blanco, Francisco Wandosell, Antonio Alcamí

**Affiliations:** 1 Centro de Biología Molecular Severo Ochoa, Consejo Superior de Investigaciones Científicas—Universidad Autónoma de Madrid, Madrid, Spain; 2 Centro de Investigaciones Biologicas en Red de Enfermedades Neurodegenerativas (CIBERNED), Madrid, Spain; Washington University, UNITED STATES

## Abstract

Herpes simplex virus type 1 (HSV-1) and HSV-2 are highly prevalent viruses that cause a variety of diseases, from cold sores to encephalitis. Both viruses establish latency in peripheral neurons but the molecular mechanisms facilitating the infection of neurons are not fully understood. Using surface plasmon resonance and crosslinking assays, we show that glycoprotein G (gG) from HSV-2, known to modulate immune mediators (chemokines), also interacts with neurotrophic factors, with high affinity. In our experimental model, HSV-2 secreted gG (SgG2) increases nerve growth factor (NGF)-dependent axonal growth of sympathetic neurons *ex vivo*, and modifies tropomyosin related kinase (Trk)A-mediated signaling. SgG2 alters TrkA recruitment to lipid rafts and decreases TrkA internalization. We could show, with microfluidic devices, that SgG2 reduced NGF-induced TrkA retrograde transport. *In vivo*, both HSV-2 infection and SgG2 expression in mouse hindpaw epidermis enhance axonal growth modifying the termination zone of the NGF-dependent peptidergic free nerve endings. This constitutes, to our knowledge, the discovery of the first viral protein that modulates neurotrophins, an activity that may facilitate HSV-2 infection of neurons. This dual function of the chemokine-binding protein SgG2 uncovers a novel strategy developed by HSV-2 to modulate factors from both the immune and nervous systems.

## Author Summary

Herpes simplex virus type 1 and 2 (HSV-1 and HSV-2, respectively) establish latency in peripheral sensory ganglia, where they remain for the lifetime of the infected individual. Understanding the mechanisms that allow these viruses to colonize the nervous system will permit devising antiviral strategies. We show that HSV-2 glycoprotein G (SgG2) binds to and increases the function of nerve growth factor (NGF), a neurotrophin expressed in the skin and mucosa essential for axonal growth and neuronal survival. This constitutes the first description, to our knowledge, of a human pathogen with the ability to augment neurotrophic factor function. The enhancement in NGF activity results in an increase in axonal growth of neurons expressing the receptor for NGF. These results were obtained *in vitro, ex vivo* and in the infected mouse, suggesting that this effect may permit a more efficient infection of NGF dependent free nerve endings by HSV-2. Absence of a similar function for HSV-1 gG may indicate a preference for the infection of particular subsets of neurons by these viruses. These results shed light on the modulation of neurotrophic factors by relevant human pathogens and on the mechanisms of colonization of the nervous system by HSV.

## Introduction

Herpes simplex virus type 1 and 2 (HSV-1 and HSV-2, respectively) are highly prevalent, neurotropic human pathogens [1]. Initial infection occurs in epithelial cells, generally within the skin and the mucosa of the oral tract and genitalia [1]. Then, HSV reaches and infects free nerve endings (FNE) of sensory neurons and colonizes ganglia of the Peripheral Nervous System (PNS). The mechanism(s) facilitating HSV neurotropism, which is crucial for latency and pathogenesis, are not well understood. Since herpesviruses are highly adapted pathogens that modify several aspects of both the immune and nervous systems, it is conceivable that they may modulate factors influencing neuronal functions to gain access to the nervous system. Several axonal guidance cues and neurotrophic factors involved in neural targeting have been identified [2]. Among them, neurotrophins are a family of secreted proteins that play relevant roles in neuronal survival, axonal growth and guidance in the PNS. Members of this family include nerve growth factor (NGF), brain-derived neurotrophic factor (BDNF), neurotrophin 3 (NT3) and NT4/5 [3]. Each neurotrophin binds with high affinity and activates tyrosine kinase receptors known as Trks. NGF binds TrkA, BDNF and NT4/5 bind TrkB, and NT3 binds TrkC. Moreover, NT3 can also bind TrkA and TrkB, although with lower affinity [3]. Both mature neurotrophins and immature precursors (proneurotrophins) also bind p75 neurotrophin receptor (p75NTR), a member of the tumor necrosis factor (TNF) receptor superfamily. p75NTR has multiple and diverse functions [4]. Another important family of neurotrophic factors is the glial cell line-derived neurotrophic factors (GDNF) family ligands (GFLs) formed by GDNF and artemin among others. GFLs interact with co-receptors of the GDNF Family Receptor α (GFRα) protein family, allowing the activation of the tyrosine kinase receptor RET (rearranged during transfection) [5]. Peripheral neurons innervating skin and mucosa show a strong dependency on neurotrophic factors both *ex vivo* and *in vivo* [6,7]. In order to colonize the PNS, HSV must reach FNE, dynamic structures capable of degeneration and regeneration [8] in response to neurotrophic factors [6,7]. The possible relevance of neurotrophic factors in the initial steps of HSV infection in neurons is not completely understood. We hypothesized that HSV could modify nerve ending navigational cues during the early steps of PNS colonization.

HSV glycoprotein G (gG) is the least conserved of the glycoproteins shared by HSV-1 and HSV-2 [9]. HSV-1 gG (gG1) and HSV-2 gG (gG2) have a similar C-terminal domain present at the virion and at the surface of the infected cells, composed by an extracellular proline-rich domain, a transmembrane region and a short cytoplasmic tail. The N-terminal domain of HSV-2 gG, but not that of gG1, is proteolytically cleaved and secreted (termed here SgG2) during infection [10,11]. Whether these differences between gG1 and gG2 have any functional consequence is unknown. Recombinant soluble gG1 (SgG1) and SgG2 bind chemokines with high affinity enhancing chemokine function [12], in sharp contrast to all previously described viral chemokine-binding proteins (vCKBPs) that inhibit chemotaxis. Since neurotrophic factors are also secreted proteins regulating many aspects of peripheral neurons, and have been previously involved in immune related-functions [13], we investigated whether HSV gG could bind neurotrophic factors modifying their activity.

Here we show that SgG2 interacts with several neurotrophic factors. SgG2, but not SgG1 or M3, a viral chemokine binding protein from murine gamma herpesvirus 68 (MHV-68) [14], transiently enhances NGF-dependent axonal growth of superior cervical ganglion (SCG) neurons *ex vivo*. The molecular mechanism beneath this enhancement involves modulation of the NGF receptor TrkA. SgG2 alters TrkA localization in lipid rafts, and NGF-dependent TrkA internalization, signaling and retrograde transport. *In vivo*, both infection with HSV-2 and expression of SgG2 in the external layers of the epidermis modifies the termination zone of the TrkA dependent FNE. Our data indicate that SgG2 may reconfigure neurotrophin signaling during HSV-2 primary infection to attract specific terminal axons to the infection site and may facilitate neural invasion.

## Results

### HSV gG interacts with neurotrophic factors

**Figure 1:**
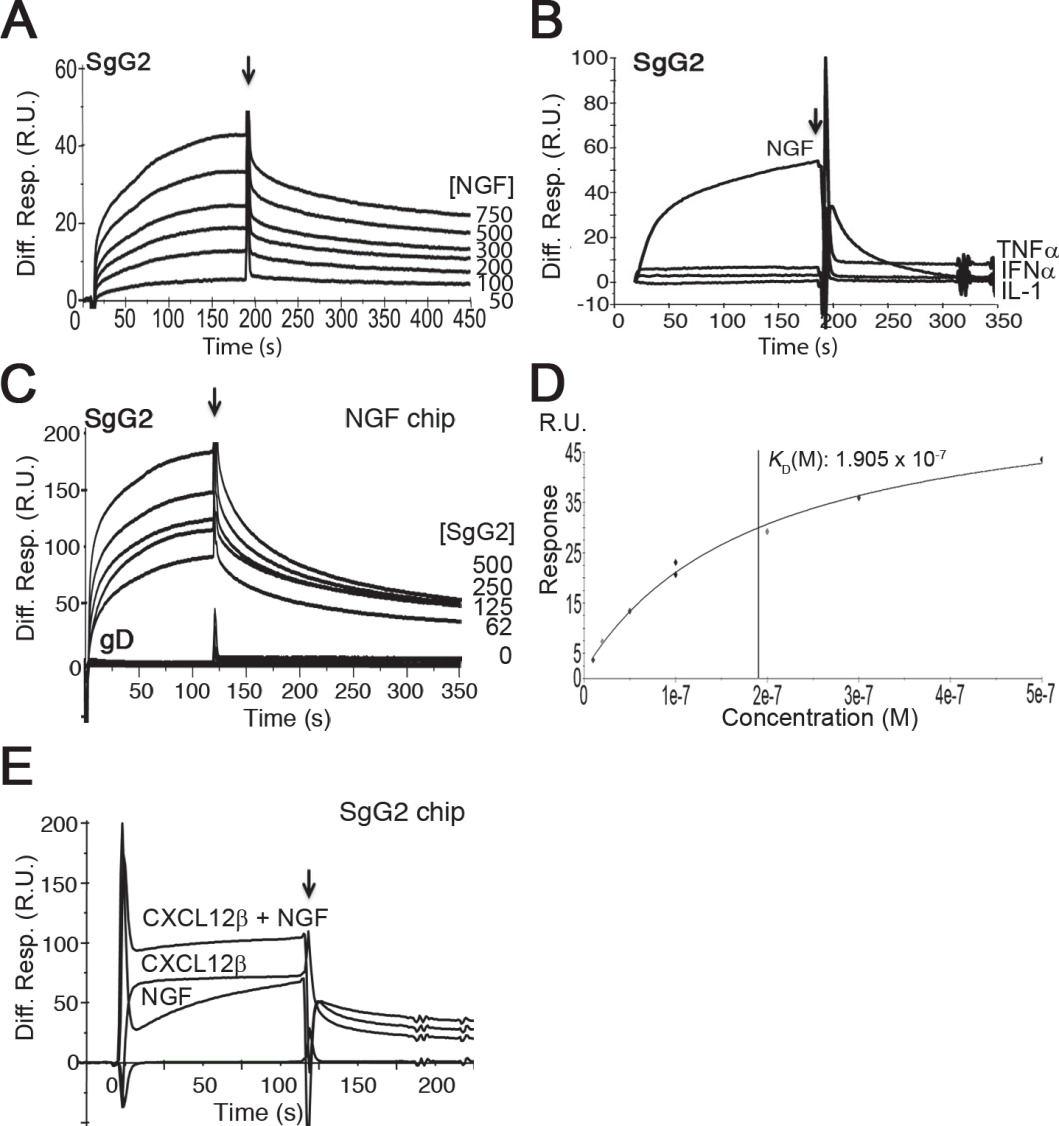
SgG2 interacts with NGF. **(A)** Sensorgram showing the interaction of SgG2 with increasing concentrations of NGF. The concentrations of NGF are indicated at the right side of the sensorgram. **(B)** Sensorgram showing the interaction of SgG2 with NGF and the lack of interaction with TNF-α, IFN-α and IL-1. All analytes were injected at a 100 nM concentration. **(C)** Sensorgram depicting the interaction between increasing concentrations of SgG2 and NGF coupled on a sensor chip. As a negative control we used the same concentrations of purified HSV-2 gD. **(D)** Saturation curve for the binding of NGF to SgG2. The derived KD is shown **(E)** Sensorgram showing the interaction of coupled SgG2 with CXCL12β and NGF injected alone or in combination. In all cases the arrow indicates the end of injection. Abbreviations: Diff. Resp., Differential response; M, molar; R.U., response units; s, seconds.

To test whether gG from HSV-1 or HSV-2 interacts with neurotrophic factors we performed surface plasmon resonance (SPR) assays. As a control we used another vCKBP from MHV-68, M3. Recombinant SgG2 interacted with members of the neurotrophin family such as NGF and those of the GFLs family, like artemin or GDNF (Fig.1A and S1 Table). SgG1 and M3 bound neurotrophic factors but saturable binding was not demonstrated in these SPR experiments and therefore non-specific binding cannot be excluded (S1A Fig. and S1 Table). However, binding specificity was suggested by the lack of interaction of the vCKBPs tested with interferon (IFN)-α, TNF-α, and interleukin (IL)-1, and the negative binding of M3 to artemin (Fig. 1B, S1B Fig. and S1 Table). Saturation experiments using SPR further demonstrated the specific, high affinity interaction of SgG2 and NGF (Fig. 1 D and S1 Table). We could also show binding of SgG1 and SgG2 to NGF coupled in a chip (S1C Fig. and Fig. 1C, respectively), with a similar affinity (K_D_ 1.4 x 10^−8^ M) to that calculated for NGF interacting with SgG2 coupled in a chip based on kinetics analysis (not shown), supporting the specificity of the interaction. HSV-2 glycoprotein D (gD) was used as a negative control for NGF binding (Fig. 1C). We confirmed binding of these vCKBPs to NGF by crosslinking assays using iodinated rat NGF ([^125^I]-rNGF) and soluble, recombinant M3, SgG1 and SgG2 (S1D Fig. and S1 Text). The formation of the vCKBP-NGF complex was competitively inhibited with increasing concentrations of unlabeled NGF (S1E, F Fig.). Addition of increasing amounts of cold NGF resulted in the formation of higher molecular weight bands that probably correspond to NGF oligomers since they appear also in the absence of viral proteins. The formation of NGF oligomers at high concentration may affect the pattern of binding to the viral proteins in our SPR assays.

Since HSV-1 and HSV-2 gG also bind chemokines, we tested whether NGF and chemokine binding takes place through different regions or whether the binding sites overlap and the interaction of NGF may be competitively inhibited with chemokines. We injected CXCL12β or NGF alone or in combination with NGF in a chip containing SgG2. The binding detected after simultaneous injection of chemokine and NGF nearly corresponded to the sum of the binding obtained when the chemokine and NGF were injected independently (Fig. 1D). Furthermore, injection of one of the analytes (NGF or CXCL12β) in an SgG2-coupled chip preincubated with the other analyte (CXCL12β or NGF) caused no displacement of the prebound analyte (not shown). It is important to note that the affinity of the interaction of SgG2 with CXCL12β (2.2x10^−9^ M) [12] is higher than that determined for NGF (2.2x10^−8^ M).

### SgG2 enhances NGF-dependent axonal growth of SCG neurons

**Figure 2:**
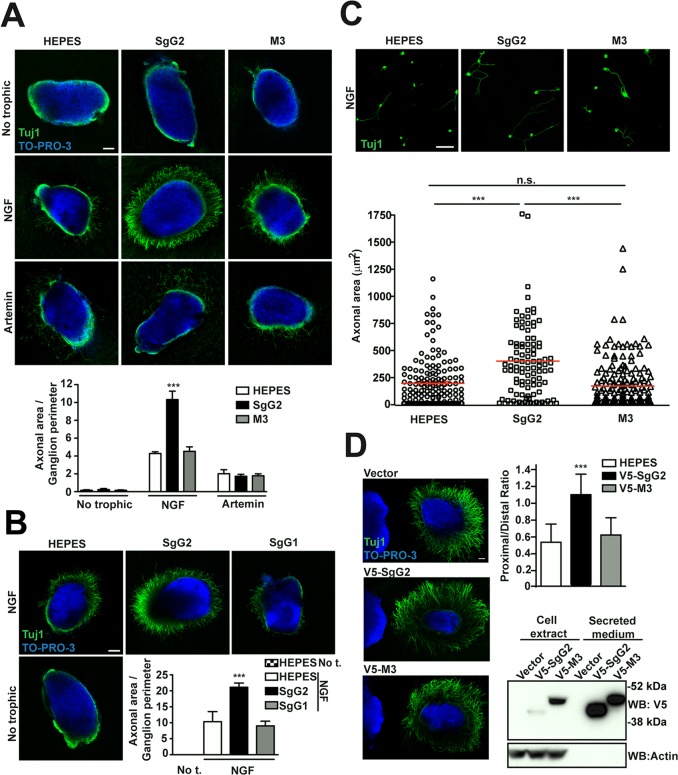
SgG2 increases NGF-dependent axonal growth. **(A,B,D)** Mouse SCGs were grown as explants in collagen matrix in the presence of the indicated trophic factors and viral proteins. Neurons were stained with Tuj1 antibody targeting class III ß–tubulin. Nuclei were stained with TO-PRO-3. Images are a projection of at least 3 stacks, correspond to the same experiment, and are representative of three **(A,D)** or two **(B)** independent experiments. The graphs below represent the quantification from three **(A,D)** or two **(B)** independent experiments. To calculate significance, a two-tailed unpaired T-test was applied. ***P<0.0001. **(A)** Explants were grown during 18 h. No trophic *n* = 7 each condition, NGF *n* = 13 each condition and artemin *n* = 9 each condition. Scale bar, 100 μm. **(B)** Explants were grown during 24 h. *n* = 7 each condition. Scale bar 100 μm. Abbreviation: No t., No trophic factor. **(C)** Mouse SCG dissociated neurons were grown during 18 h in the presence of NGF and viral proteins as indicated. Images are a projection of at least 5 stacks and correspond to the same experiment. HEPES *n* = 172, SgG2 *n* = 103 and M3 *n* = 181. Scale bar 50 mm. The graph shows the quantification of the axonal area for each condition. **(D)** Hanging drops containing transfected HEK-293T (on the left side of the images) were placed in the proximity of SCG explants and grown during 48 h with NGF. *n* = 15 for each condition. Scale bar 100 μm. The bottom right panel shows the expression and secretion of viral proteins in transfected HEK293T cell by western blot using an anti-V5 antibody.

We addressed whether the vCKBP could affect the function of neurotrophic factors. FNE of sensory neurons are the main targets of HSV-1 and HSV-2 in the skin and mucosa. We focused on NGF and artemin due to their relevance in epidermal homeostasis and innervation [6,7]. In order to have a feasible and robust model we used mouse sympathetic neurons from SCG that express high levels of NGF and artemin receptors, and depend on NGF and artemin both *in vivo* and in culture [15]. We cultured SCG as 3D explants using collagen matrix containing recombinant viral proteins, and we measured the area comprised by axons normalizing to the ganglion perimeter. In the presence of NGF, SgG2 enhanced axonal growth of SCG neurons compared to HEPES control (Fig. 2A, middle panels). On the contrary, M3 (Fig. 2A, middle panels) did not induce such increase. No changes in axonal growth induced by SgG2 or M3 were detected in the absence of trophic factors (Fig. 2A, upper panels), or when artemin was used (Fig. 2A, lower panels). Next, we addressed whether SgG1 had the same effect on NGF as SgG2. SgG1 did not significantly increase axonal growth of SCG neurons 24 h post-incubation (Fig. 2B). SgG2 enhancement of NGF-mediated axonal growth was also observed when using dissociated SCG neurons (Fig. 2C). To further characterize this effect and to discriminate between axonal growth and directionality, transfected HEK-293T cells were co-cultured with SCG in 3D explants with NGF, and the proximal/distal (P/D) ratio was analyzed. HEK-293T cells express endogenous axonal repulsive cues like semaphorin 3A [16] and several class-A ephrins [17]. Accordingly, using low concentrations of NGF (0.25 nM), control transfected HEK-293T cells induced repulsion of SCG axons (Fig. 2D). Such repulsion was also observed when HEK-293T cells were transfected with a V5-tagged M3-expressing plasmid (V5-M3). On the contrary, the expression of V5-SgG2 significantly reduced the repulsion of SCG axons (Fig. 2D middle panel). Viral protein expression was detected by Western blot (Fig. 2D). Altogether, these data showed that SgG2 specifically increases axonal growth of SCG neurons in an NGF-dependent manner.

### SgG2 modifies NGF-TrkA signaling

**Figure 3:**
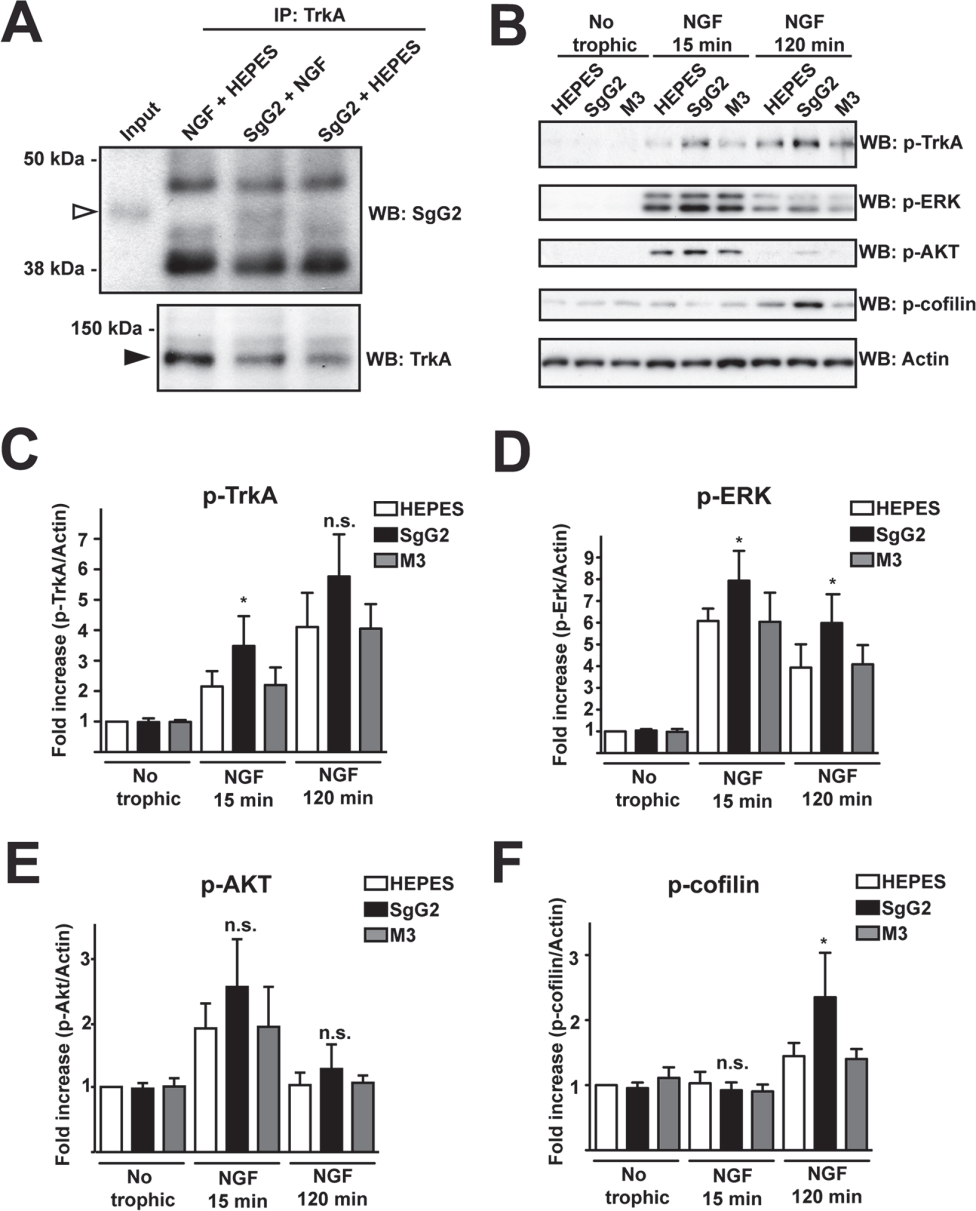
SgG2 modifies NGF-TrkA signaling. Mouse SCG dissociated neurons were grown during 5 days *in vitro* (DIV). **(A)** Neurons were deprived of NGF for 16 h and were stimulated with NGF alone, NGF plus SgG2 or SgG2 alone during 5 min. TrkA-SgG2 interaction was analyzed by TrkA immunoprecipitation (IP) followed by Western blot (WB) to detect SgG2. Molecular sizes in kDa and the position of SgG2 (empty arrowhead) and TrkA (solid arrowhead) are indicated. The experiment shown is representative of three independent assays. **(B-F)** Neurons were deprived of NGF for 16 h and were stimulated with NGF and the indicated viral proteins during 0, 15 and 120 min. **(B)** The phosphorylation levels of TrkA, ERK, AKT and cofilin were analyzed by Western blot (WB) using specific antibodies. Detection of actin was used as a loading control. All blots correspond to the same experiment. Graphs show statistical analysis for **(C)** p-TrkA, **(D)** p-ERK, **(E)** pAKT and **(F)** p-cofilin levels. p-TrkA *n* = 4; p-ERK *n* = 4; p-AKT *n* = 4; p-cofilin *n* = 3. To calculate significance a two-tailed unpaired T-test was employed; *P<0.05; n.s: not significant.

We addressed whether the increase in NGF-dependent axonal growth mediated by SgG2 was related to changes in TrkA. Since SgG2 bound NGF and modulated its function we decided to address whether it could interact with TrkA. We incubated dissociated SCG neurons with NGF in the presence or absence of SgG2. Following immunoprecipitation of TrkA, SgG2 was detected only when NGF was present, indicating that SgG2 and TrkA belong to the same complex in the presence of NGF and the interaction of SgG2 with NGF is required (Fig. 3A). Then we focused on TrkA signaling. Dissociated cultures of SCG neurons were incubated with NGF, M3 or SgG2 alone or NGF together with the viral proteins, and the downstream signaling was analyzed by Western blotting. Incubation with SgG2 or M3 in the absence of NGF did not induce TrkA phosphorylation or activation of downstream pathways (Fig. 3B–F). As expected, NGF promoted TrkA phosphorylation [3]. SgG2 significantly increased NGF-dependent TrkA phosphorylation at tyrosine 490 (Tyr490) at 15 min, but not at 120 min post-incubation, when compared to control and M3 conditions (Fig. 3B,C). Extracellular signal-regulated kinases (ERK)1/2 activation in response to NGF, but not that of AKT (also known as protein kinase B), was significantly increased in the presence of SgG2 both at 15 and 120 min post-incubation (Fig. 3B,D,E). We also tested the activation status of cofilin, an actin-severing protein responsible of actin turnover that is inactivated by phosphorylation [18]. We detected higher levels of cofilin phosphorylation induced by SgG2-NGF 120 min post-NGF stimulation when compared to NGF alone (Fig. 3B,F).

### SgG2 alters localization of TrkA in lipid rafts

**Figure 4:**
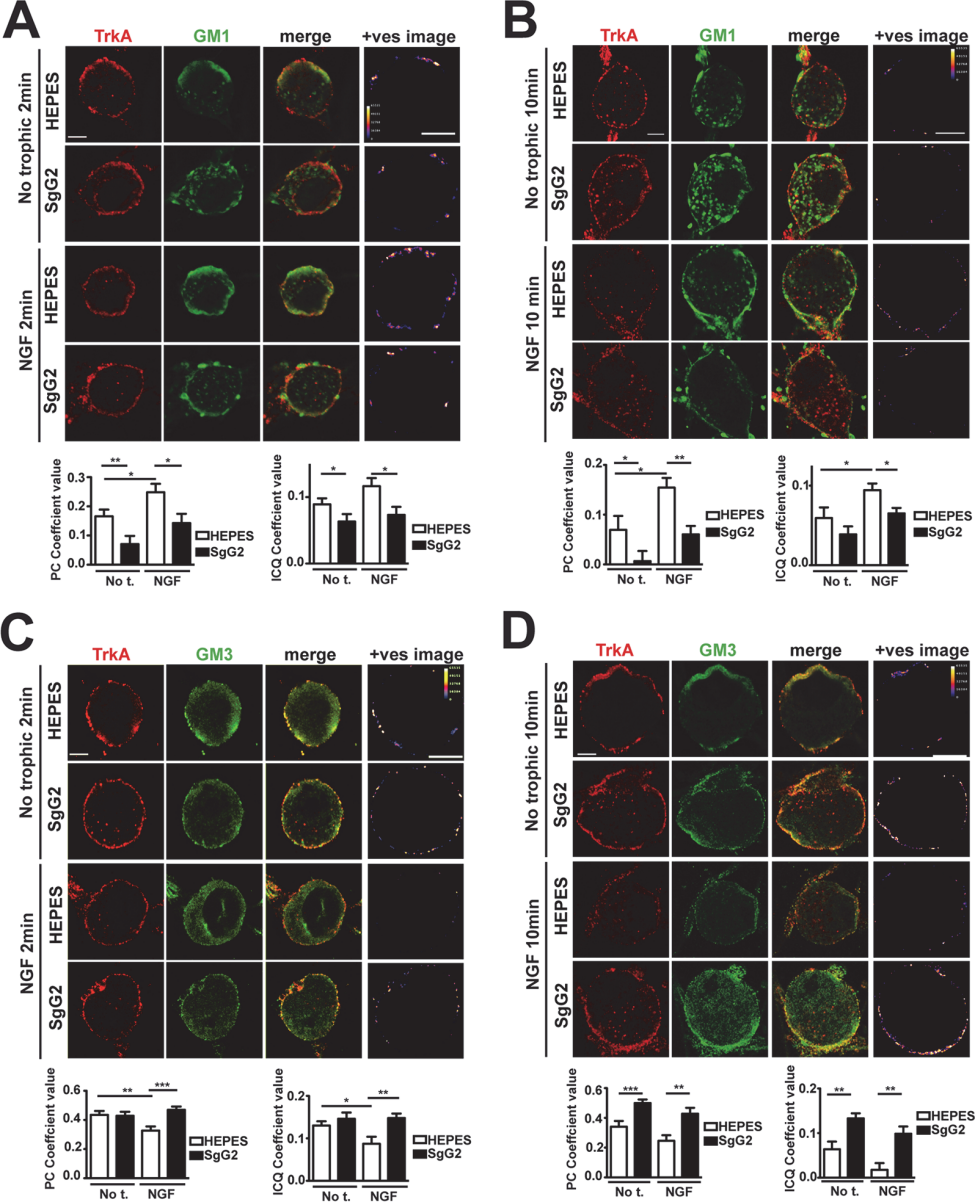
SgG2 promotes the incorporation of TrkA into different microdomains of the plasma membrane. Mouse SCG dissociated neurons were grown during 5 DIV. Neurons were deprived of NGF for 16 h and subsequently stimulated with NGF, vCKBPs or both. Colocalization between TrkA and two different subtypes of lipid rafts was studied using immunofluorescence without permeabilization. TrkA colocalization with GM1 rafts 2 min **(A)** and 10 min **(B)** post-stimulation. TrkA colocalization with GM3 rafts 2 min **(C)**, and 10 min **(D)** post-stimulation. Confocal microscopy images correspond to one representative cell from each condition. The +ves image displays pseudocolored pixels from the areas within the plasma membrane in which both TrkA and the corresponding subtype of lipid rafts pixel value exceed the mean. Scale bar represents 5 μm. Graphs show the average values (mean±SEM) obtained for PC and ICQ. Plots in **(A)** represent *n* = 18–26 neurons from three independent assays. Plots in **(B)** show the results obtained for 15 cells from two independent experiments. Plots in **(C)** represent *n* = 21–35 neurons from three independent assays. Plots in **(D)** represent 16–23 neurons from two independent assays. Two-tailed unpaired T-test, *P<0.05; **P<0.001; ***P<0.0001.

At the cell surface there are different and segregated types of rafts depending on their lipid and glycosphingolipid composition, leading to diverse biological functions [19]. A fraction of TrkA accumulates in GM1 lipid rafts, and TrkA appears to signal through GM1 rafts [20,21]. We addressed whether SgG2 could affect TrkA recruitment to GM1-enriched lipid rafts in SCG dissociated neurons. In the absence of NGF, a small fraction of TrkA co-localized with GM1 staining (Fig. 4A, top row). As predicted, NGF stimulation maintained and increased TrkA co-localization with GM1 staining 2 min post NGF stimulation (Fig. 4A, third row). SgG2 disrupted NGF-dependent and-independent TrkA localization in GM1 rafts at 2 (Fig. 4A, fourth row) and 10 min post-stimulation (Fig. 4B, fourth row). We hypothesized that SgG2 could divert TrkA to other types of rafts such as GM3 rafts [19]. Stimulation with NGF reduced the localization of TrkA in GM3-rich rafts (Fig. 4C,D, third row) concomitantly with NGF-dependent TrkA increase in GM1 rafts, when compared to HEPES treatment. However, addition of SgG2 retained TrkA within GM3 rafts at 2 min post-incubation (Fig. 4C, fourth row) and even more at 10 min post-incubation (Fig. 4D, fourth row). Overall, our results indicated that SgG2 recruits TrkA to GM3-enriched lipid rafts, thereby altering the normal NGF-TrkA signaling that usually occurs within GM1 rafts.

The NGF receptor p75NTR is present in GM1 rafts [20] and interacts with TrkA in an NGF-dependent manner [3,22]. We addressed the effect of SgG2 on the interaction between TrkA and p75NTR. As shown in S2 Fig., SgG2 disrupts the NGF-induced TrkA-p75NTR interaction in agreement with TrkA raft relocation mediated by SgG2, whereas SgG1 did not. As TrkA recruitment to GM3 appeared to be independent of the presence of NGF in the culture we wanted to determine whether SgG2 alone was sufficient to induce changes in TrkA signaling. To explore this, we preincubated dissociated SCG neurons with HEPES or with SgG2 for 10 min followed by NGF stimulation in the presence or absence of SgG2. Preincubation with SgG2 did not affect NGF dependent TrkA or ERK phosphorylation (S3A–C Fig.) and, in agreement with Fig. 3B, changes on signaling were detected only when SgG2 was added together with NGF. These results indicated that SgG2 induces TrkA raft relocalization that may be necessary, but not sufficient, to enhance NGF-TrkA signaling.

### SgG2 partially blocks NGF-dependent TrkA internalization and retrograde transport

**Figure 5:**
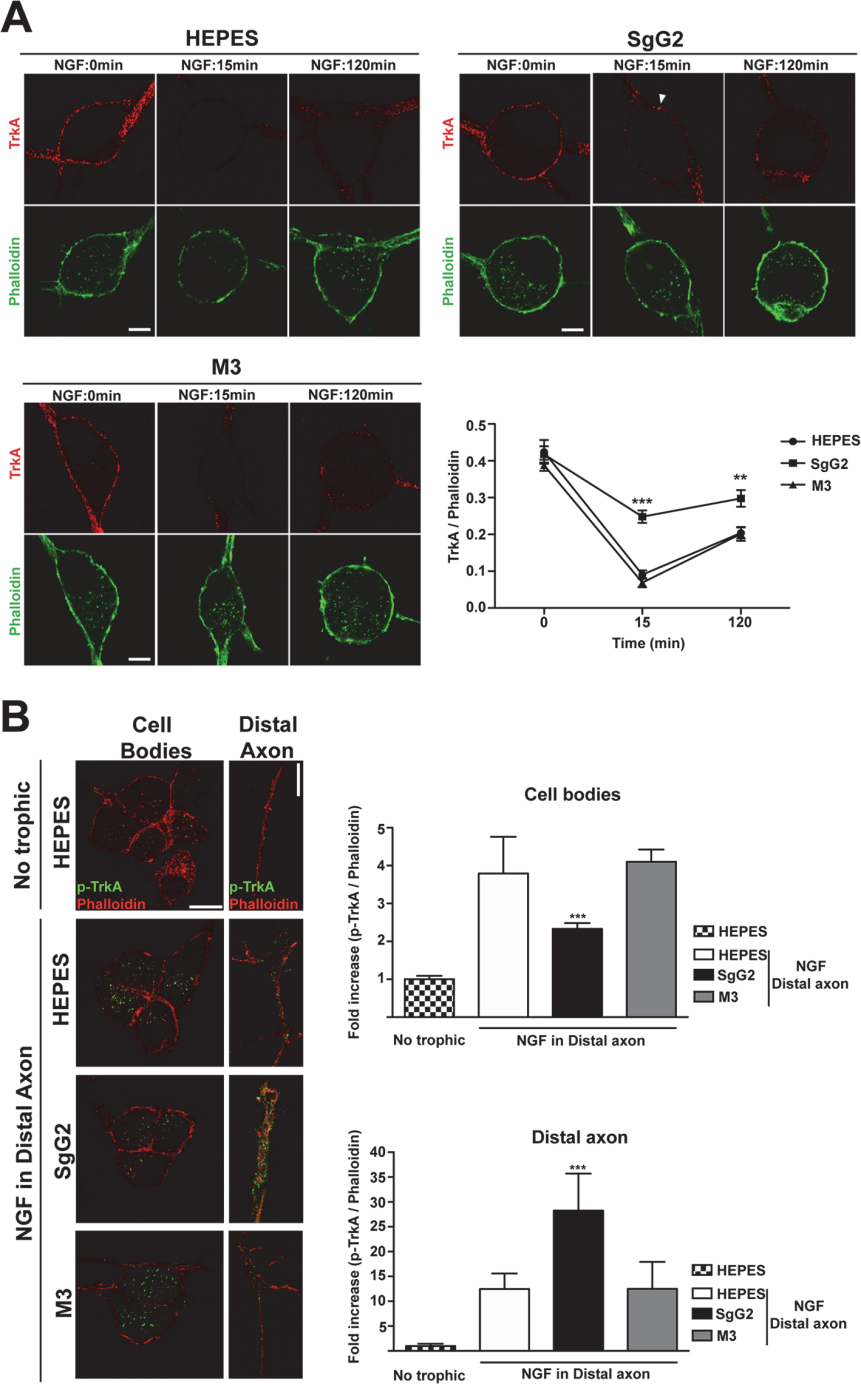
SgG2 reduces NGF induced TrkA internalization and retrograde transport. SCG dissociated neurons were grown during 5 DIV and deprived of NGF for 16 h. **(A)** Neurons were stimulated with NGF alone or together with the indicated viral proteins during 0, 15 and 120 min. TrkA internalization was analyzed by immunofluorescence without permeabilization and normalized for F-actin using phalloidin staining. Solid arrowhead points to a non-internalized cluster of TrkA at the plasma membrane. Images show a projection of at least 3 image planes. All images correspond to the same experiment, representative of two independent experiments (*n* = 12 for each condition). Scale bar represents 5 μm. The graph shows the quantification of the TrkA/phalloidin staining at the plasma membrane over time in samples treated with NGF plus the indicated protein or buffer. **(B)** Mouse SCG dissociated neurons were grown during 7 DIV using microfluidic devices. Neurons were deprived of NGF for 16 h and NGF and viral proteins were added to the distal axon compartment during 120 min. TrkA phosphorylation was analyzed by immunofluorescence after permeabilization with Triton X-100 and normalized using phalloidin. The images and the graph correspond to the same experiment and are representative of three independent experiments. Scale bar 10 μm. *n* = 10 fields for each condition. A two-tailed unpaired T-test was used to calculate significance in **(A)** and **(B)**. ***P<0.0001.

Careful examination of the results obtained following 10 min of NGF incubation (Fig. 4B,D) suggested a possible effect of SgG2 at the level of plasma membrane TrkA. To confirm this observation we analyzed the internalization rate of TrkA in response to NGF and SgG2. Similar amounts of TrkA were detected in almost every experimental condition in the absence of NGF (Fig. 5A, time 0 min). Addition of 1 nM NGF promoted maximal TrkA internalization at 15 min post NGF incubation in both control (HEPES) and M3 conditions (Fig. 5A). However, when SgG2 and NGF were added simultaneously we observed higher levels of TrkA at the plasma membrane (Fig. 5A). Following 120 min of NGF exposure, this difference was maintained although TrkA at the neuronal surface increased both in control and M3 conditions, probably due to receptor recycling (Fig. 5A). *In vivo*, neurotrophins are secreted by specific tissues and only the axon terminals are directly exposed to them [23]. For a suitable signaling on neurons to occur, neurotrophins must be transported in a retrograde manner from distal axons to their cell bodies. TrkA internalization seems to be required for TrkA retrograde transport [23]. Cofilin is a critical mediator of TrkA retrograde transport [18]. Stimulation of SCG neurons with NGF and SgG2 induced significant higher levels of cofilin phosphorylation, resulting in its inactivation [24], at late time points (Fig. 3). We addressed the effect of SgG2 on TrkA retrograde transport by monitoring the localization of phosphorylated TrkA (p-TrkA) on SCG neurons, grown in microfluidic devices, whose axon terminals were incubated with NGF alone or in combination with vCKBPs during 120 min. We found very low levels of p-TrkA in the distal axons or cell bodies of neurons not exposed to NGF (Fig. 5B, upper row). As expected, exposure of distal axons to NGF during 120 min promoted a moderate increase on p-TrkA staining in distal axons and a high increase of p-TrkA staining in the cell bodies (Fig. 5B, second row). Similar results were obtained when distal axons were exposed to NGF and M3 (Fig. 5B, fourth row). Exposure of distal axons to NGF and SgG2 induced a significant accumulation of p-TrkA staining in distal axons whereas low level staining was present in the cell bodies (Fig. 5B, third row and graph). Moreover, the growth cone had a normal morphology following 120 min exposure to NGF in both control and M3 conditions, with large actin-rich filopodial protrusions. The presence of SgG2 severely disturbed growth cone morphology, presenting only few filopodia (NGF-HEPES: 88,8% spread growth cones, NGF-M3 94,7% spread growth cones and NGF-SgG2: 6,2% spread growth cones; *P* = 0,00303681, *P* = 0,00052529, respectively). Altogether, these data indicated that SgG2 partially blocks NGF-dependent TrkA endocytosis and retrograde transport with p-TrkA accumulating at the distal axon in the presence of NGF-SgG2. Also, the actin cytoskeleton is perturbed.

### SgG2 modifies peptidergic FNE termination zone in mouse epidermis

**Figure 6:**
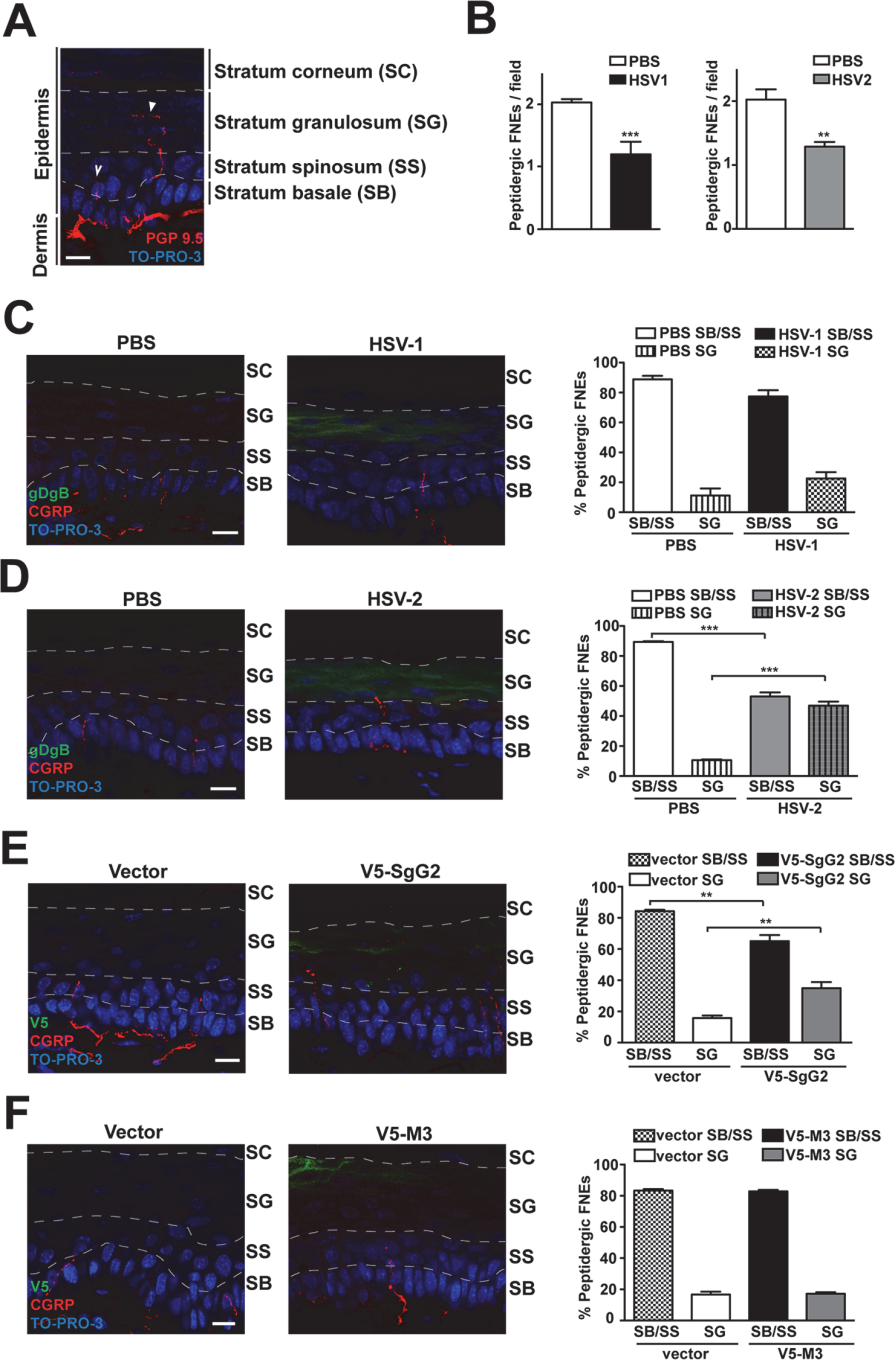
V5-SgG2 transfection in the epidermis modifies the termination site of peptidergic FNE. **(A)** Image of FNE in the mouse epidermis stained with anti-PGP 9.5, a pan-neuronal marker. The image shows the different morphologies of the FNE. Solid arrowhead points to a FNE with morphology that fits with the majority of the non-peptidergic FNE. Open arrowhead points to a FNE with morphology that fits with the majority of the peptidergic FNE. **(B)** Graph representing the number of peptidergic FNE per field in infected and non-infected mouse hindpaw epidermis. **(C, D)** Mouse hindpaw epidermis was treated with PBS (right hindpaw) or infected with HSV-1 **(C)** or HSV-2 **(D)** (left hindpaw) and stained with and anti-gBgD antibody to detect HSV and the anti-CGRP antibody to detect the peptidergic FNE. Termination zone of FNE was determined and quantified. Images show the projection of at least 3 planes. 4 mice were infected and 15 fields were analyzed for each condition. **(E, F)** Mouse hindpaw epidermis was transfected with empty vector (right hindpaw) or with a V5-SgG2 **(E)** or V5-M3 **(F)** construct (left hindpaw) and stained with the peptidergic marker antibody anti-CGRP and anti-V5 epitope. Termination zone of FNE was determined and quantified. Images show the projection of at least 3 planes. The experiment shown is representative of 3. 15 fields were analyzed for each condition. In **(A-F)** the discontinuous white lines are shown to indicate the separation between the different layers of the epidermis. Scale bar 10 μm. A two-tailed unpaired T-test was used to calculate significance. **P<0.001; ***P<0.0001.

We hypothesized that SgG2-modification of NGF function could attract axons to infected sites *in vivo*. NGF is secreted by keratinocytes in the skin [6]. In adult mice glabrous skin there are two morphologically different intra-epidermal FNE, with distinct termination zones ([25], Fig. 6A): (i) FNE that reach and meander through the *stratum granulosum*, an external layer of epidermis (Fig. 6A, solid arrowhead), most of them being non-peptidergic and expressing RET; and (ii) FNE with straight trajectory that remain in the inner *stratum basale* or *stratum spinosum* (Fig. 6A, open arrowhead), most of them being from peptidergic TrkA^+^ neurons [25–27].

Since SgG2 specifically modifies NGF-dependent TrkA^+^ signaling, we analyzed peptidergic FNE *in vivo* during HSV-1 or HSV-2 infection. We topically applied PBS, HSV-1 or HSV-2 onto mice hindpaw (glabrous skin) following mild skin exfoliation. We did not detect any macroscopical difference in the skin between the different conditions 48 h post-infection. We detected HSV infection with an anti-gBgD antibody and the peptidergic FNE using an anti-calcitonin gene related peptide (CGRP) antibody. The number of peptidergic FNE was significantly reduced in skin infected with both HSV-1 and HSV-2 (Fig. 6B). Most peptidergic FNE remained in the *stratum basale* or in the *stratum spinosum* in PBS-treated skin, as described [25] (Fig. 6C left panel, 6D left panel). HSV-1 infection promoted a subtle, although not significant, change in the trend of the termination zone of the remaining peptidergic FNE (Fig. 6C, right panel). However, following HSV-2 infection nearly half of the remaining peptidergic FNE increased in length, reaching the *stratum granulosum* (Fig. 6D, right panel).

We hypothesized that changes in straight peptidergic FNE termination zone induced by HSV-2, but not by HSV-1, could be mediated by SgG2 through its interaction with NGF. We transfected mice hindpaw skin with empty vector or viral cDNA coding for V5-SgG2 or V5-M3. Transfection did not affect epidermal layers, general nerve arrangement or the number of peptidergic FNE per field in all the conditions tested. In vector-transfected skin, nearly all straight trajectory-peptidergic FNE remained in the *stratum basale* or in the *stratum spinosum*, (Fig. 6E, left panel, 6F left panel). However, in the fields where V5-SgG2 expression was detected, around 20% of straight trajectory-peptidergic FNE showed increased length, reaching the *stratum granulosum* (Fig. 6E, right panel). This phenomenon was not detected when V5-M3 was expressed (Fig. 6F, right panel). Altogether these data suggested that HSV-2, but not HSV-1, modifies the termination zone of the straight peptidergic FNE and that SgG2 may account, at least partially, for this phenomenon.

## Discussion

Neurotropism is a major evolutionary advantage for HSV. The infection of neuronal FNE permits HSV to establish latency in sensory ganglia and persist for the lifetime of the individual. We have detected that infection of glabrous skin with HSV-1 and HSV-2 causes a reduction in the number of peptidergic FNE. This could be due to the secretion of neurotoxic molecules following infection, such as IL-1β and TNF-α [28,29]. HSV may have developed molecular mechanisms to counteract this toxicity. The presence, plasticity and topology of FNE during development and adult remodeling depend on axonal navigational cues and trophic factors like neurotrophins. Our results show that HSV-2 SgG specifically binds to several neurotrophic factors from the neurotrophin family (NGF, BDNF and NT3) and the GFL family (GDNF and artemin). Interactions of HSV-1 gG and MHV-68 M3 with neurotrophic factors were also observed but we cannot rule out the possibility that these interactions are non-specific. Further research may identify biological relevance for these interactions in the biology of HSV-1 and MHV-68. Only the interaction between SgG2 and NGF is biologically relevant in our experimental model resulting in an increase in FNE growth of TrkA^+^ neurons. This constitutes the first description, to our knowledge, of a protein expressed by a human pathogen with the ability to modulate neurotrophic factors and we hypothesize that this interaction may contribute to HSV neurotropism.

To test this hypothesis we focused our functional studies on neurotrophic factors regulating innervation of the epidermis and that are important in skin homeostasis and inflammation, like NGF and artemin [6,7,13]. Using SCG explants and dissociated cultures, only SgG2, but not SgG1 or M3, modified NGF-dependent axonal growth *ex vivo*. *In vivo* only SgG2 modified the growth and termination site of the TrkA^+^ FNE, corresponding to peptidergic neurons. Importantly, similar results were observed when infecting the skin with HSV-2 but not with HSV-1.

One of the most intriguing results of our work is the difference between HSV-1 and HSV-2. HSV-1 seroprevalence is higher than that of HSV-2 [30–32]. HSV-1 is normally transmitted during childhood and is linked to facial herpes whereas HSV-2 is normally sexually transmitted and associated with genital herpes. However, both viruses can infect these two anatomical areas, and genital herpes due to HSV-1 is increasing [31,33]. Of note, HSV-1 reactivates in the genital tract less frequently than HSV-2 [34]. Therefore, differences in prevalence, transmission route and recurrent disease between HSV-1 and HSV-2 in the oro-labial or genital area exist. The lack of effect with HSV-1 in our *in vivo* experiments does not rule out the possibility that HSV-1 may modulate neurotrophic factors in another setting or utilizes a different mechanism to modulate axonal growth. In this regard, HSV-1 latency associated transcript induces axonal regeneration and growth in a post-entry phase following serum deprivation [35] or NGF starvation [36], and this could facilitate release of HSV-1 into the peripheral tissue following anterograde transport from the neuronal cell body [36]. From a molecular perspective, the different activity of gG1 and gG2 could be due to their structure and location. HSV-1 gG is a transmembrane protein that remains anchored at the surface of infected cells and the virion envelope whereas gG2 is proteolytically processed secreting an N-terminal domain, SgG2, with the potential of reaching neighboring cells.

Mature peptidergic neurons express TrkA. These type of FNE represent 40% of the total FNE in glabrous skin [25], but they are the predominant population in mucosa and in the internal organs [37,38]. The fact that HSV-2 displays a specific molecular mechanism involved in attracting peptidergic FNE points to possible implications on a more efficient colonization of different anatomical niches or subsets of neurons. In this regard, recent evidence indicates that sensory A5^+^ neurons (corresponding to CGRP^+^/TrkA^+^ neurons) support HSV-2 productive infection while they are non-permissive for HSV-1 productive infection *in vitro* [39]. This difference in susceptibility is dependent on the latency associated transcript [40]. Whether the lack of HSV-1 effect on TrkA^+^ FNE shown here may influence A5^+^ neuron susceptibility requires further investigation. The genitalia are enriched in TrkA-expressing peptidergic innervation [41,42]. For instance, TrkA^+^ projecting neurons rise up to 60% from first sacral ganglia to rat penis [43]. We propose that SgG2 may facilitate the infection of FNE innervating the genitalia and/or subsequent spread. In agreement with this hypothesis, pharmacological modulation of capsaicin-sensitive peptidergic neurons reduces the severity of cutaneous HSV-2 genital infections both in female and male pigs [44]. Alternatively, infection of peptidergic neurons may be relevant for nociception. In summary, we cannot conclude from our study that HSV-2 SgG-mediated modulation of NGF is essential for the infection of FNE and subsequent colonization of the nervous system, but our data lead us to propose that SgG2 may facilitate the infection of specific subsets of neurons and this may have consequences for transmission and disease. Deletion or disruption of *us4*, the gene encoding gG, in HSV-1 results in partial attenuation in mouse models of infection [45–47]. There are no reports analyzing the role of gG2 *in vivo* using animal models.

Transient enhancement of NGF-mediated axonal growth by SgG2 involves several related events affecting TrkA localization and trafficking. SgG2 alone modifies TrkA association with gangliosides, increasing its relative presence in GM3- versus GM1-rich rafts. This effect could be mediated by the SgG2 ability of binding glycosaminoglycans (N. M.-M., A. V.-B. and A. A. submitted manuscript)). However SgG2 mediated TrkA recruitment to GM3 is not sufficient to induce changes in TrkA signaling by itself. SgG2 must be bound to NGF to promote the described changes as previously observed for SgG2 enhancement of chemokine function [12]. GM1 is the preferential ganglioside for canonical NGF-TrkA signaling [21]. GM1 is a marker of caveolae and caveolae-like membranes [20]. The SgG2-mediated TrkA exclusion from GM1 rafts may influence the function of TrkA and could explain the reduced TrkA-p75NTR interaction upon NGF stimulation since p75NTR is located mainly within caveolae and caveolae like membranes [20]. TrkA-p75NTR interaction appears to be required for NGF-TrkA endocytosis in some models [22] and could account at least partially for the reduced TrkA endocytosis detected in the presence of SgG2 during NGF stimulation. The fact that TrkA is a raft resident protein [48], whereas RET remains outside lipid rafts and is transiently recruited to rafts upon binding to GFLs in cis [49,50], could explain the lack of SgG2 effect on Artemin function. Since TrkA endocytosis is a pre-requisite for its retrograde transport [18,51], this could explain the blockage of NGF-mediated TrkA retrograde transport caused by SgG2. Finally, accumulation of p-TrkA in distal axons mediated by NGF-SgG2 could induce a local increase in axon length as proposed for NT-3 activation of TrkA [51,52]. The SgG2-NGF-TrkA complex promotes an aberrant downstream signaling. The enhanced NGF-mediated TrkA phosphorylation in the presence of SgG2 could be due to the presence of higher levels of TrkA at the plasma membrane, to differences in NGF-TrkA sensitivity, or to the modification of TrkA-ganglioside association since the raft environment determines the interaction of receptors with specific signaling components [19]. One of the most intriguing questions that arise from SgG2-NGF-TrkA signaling resides in the long-term inactivation of cofilin through phosphorylation. Cofilin is an actin-severing protein that regulates actin turnover [24]. Besides, cofilin is a key component of the TrkA retrograde transport complex [18]. Our data showed that SgG2 blocks NGF-induced TrkA retrograde transport and disturbs growth cone morphology. Both results are in agreement with the observed phosphorylation of cofilin. An increased amount of p-TrkA in distal axons may lead to a rapid and transient axonal growth. Growth cones appear to function as a probe [53], and exposure to SgG2-NGF reduces filopodia resulting in a blunt growth cone. These blunt growth cones could be less responsive to repulsive navigational guidance cues and, together with an increased TrkA and ERK signaling, may reach distant, non-permissive sites. All these molecular events promoted by SgG2, raft relocation of TrkA, reduced internalization of TrkA in response to NGF and activation of modified NGF signaling pathway, appear to be interconnected finally resulting in the invasion of external epidermal layers by peptidergic FNE when SgG2 is ectopically expressed in mouse footpad skin, probably facilitating HSV-2 access to this type of FNE.

We have shown that HSV gG is a vCKBP with the unique property of enhancing the activity of chemokines [12], immune proteins involved in the migration and activation of leukocytes [54]. Both gG1 and gG2 potentiate chemokine-mediated migration *in vitro* and *in vivo*, and this may facilitate the infection of cells recruited to areas of infection and thereby virus dissemination to other tissues. The migration of uninfected epithelial cells towards HSV infected sites has been shown [55] supporting the ability of HSV to modulate cell migration. Here we demonstrate that HSV SgG2 interacts with neurotrophic factors and enhances NGF-dependent growth of FNE to sites of infection probably facilitating transmission to the PNS. Interestingly, SgG2 uses a common mechanism to enhance chemokine and NGF activity, causing the relocalization of their receptors to specific ganglioside-rich sites in the plasma membrane and delaying the internalization rate of the receptors, increasing their levels at the cell surface (this report and N. M.-M., A. V.-B. and A.A., manuscript submitted). SgG2 seems to bind both chemokines and NGF simultaneously and this property could play a relevant role during *in vivo* infection due to the contribution of chemokines and neurotrophins in the crosstalk between the immune and nervous system. Chemokines are essential in the antiviral response but they can also modulate the responsiveness of axons to guidance cues, acting as antagonists of axonal repulsion and inducing axonal sprouting [56–58]. Neurotrophic factors are essential elements of the nervous system and also play relevant roles in inflammation and pain modulation [59]. NGF is the major contributor of axonal growth of peptidergic neurons and a key component of the neurogenic inflammatory response in the epidermis in response to stress or under pathological conditions, permitting a coordinated response between immune cells and peptidergic neurons [60–62]. Our results *in vitro*, *ex vivo* and *in vivo*, lead us to propose that SgG2 modifies NGF-TrkA signaling to induce peptidergic neurons to grow to external layers of the epidermis, facilitating access of HSV-2 to peptidergic neurons.

This is, to our knowledge, the first example of a viral protein, encoded by a highly prevalent neurotropic human pathogen that interacts with components of both immune and nervous systems, and both activities may help HSV-2 to reach FNE to establish latency in neurons. A similar strategy may be used by other relevant human pathogens. The interaction of SgG2 with neurotrophic factors sheds light on the complex network of virus-host interactions and uncovers a new molecular framework to investigate the colonization of the nervous system by HSV-2, an important human pathogen.

## Materials and Methods

### Ethics statement

All animal experiments were performed in compliance with national and international regulations and were approved by the Ethical Review Board of the Centro de Biología Molecular Severo Ochoa under the project number SAF2009–07857. The procedures employed complied with the National (“Real Decreto” 1201/2005 and 53/2013) and European (Directives 86/609/CEE and 53/2013) regulations.

### Determination of vCKBP-neurotrophic factor binding specificity and affinities

The interactions between neurotrophic factors and vCKBPs were characterized by SPR technology as before [63], with Biacore X and X100 biosensors (GE Healthcare). SgG1, SgG2 and M3 were coupled to CM4 or CM5 Biacore chips through amine coupling. In the screening experiment, to determine binding to SgG1, SgG2 and M3, neurotrophic factors (Peprotech) were injected at 100 nM in HBS-EP buffer (10 mM HEPES, 150 mM, NaCl, 3 mM EDTA, 0.005% (vol/vol) surfactant P20, pH 7.4) at a flow rate of 10 μl/min, and association and dissociation were monitored. Other analytes used (IFN-α, TNF- α and IL-1, Peprotech) were also injected at 100 nM. To determine the association and dissociation kinetics, different concentrations of neurotrophic factors (with the exception of artemin) were injected at a flow rate of 30 μl/min, and association and dissociation were monitored. The number of relative units in each chip was 831 (for SgG1), 187 (for SgG2) and 375 (for M3). Artemin was coupled to a CM5 chip and different concentrations of SgG1, SgG2 and M3 were injected. Human NGF was coupled to a CM4 chip and different concentrations of SgG1, SgG2 and HSV-2 gD were injected. In some experiments NGF and CXCL12β were injected alone or in combination into a chip containing SgG2 to address whether SgG2 could bind both simultaneously. All Biacore sensorgrams were analyzed with the software Biaevaluation 3.2 and Evaluation 2.0.1 (for sensorgrams obtained with the Biacore X and Biacore X100, respectively)

### Culture of explants of SCG in collagen matrix

Mouse sympathetic SCG were cultured in a 3D collagen matrix [64]. In brief, 340 μL of rat tail collagen I (BD Biosciences, San Jose, CA) were mixed with 40 μL of 10x MEM, 10 μL of HEPES or the vCKBPs at a final concentration of 50 nM, and mouse NGF 2.5S (N-100, Alomone labs, Jerusalem, Israel) at a final concentration of 0.25 nM. This mix was neutralized using 0.8 M NaHCO_3_, and immediately spotted in drops where ganglia were included.

### Culture of dissociated SCG neurons

Mouse sympathetic neurons from SCGs were cultured as previously described [65]. Ganglia were dissected from newborn mice (postnatal day 0–1), digested in collagenase and trypsin (Worthington, Lakewood, NJ), dissociated by trituration and plated on dishes previously coated with rat tail collagen I (BD Biosciences) in DMEM containing 50 ng/mL NGF (Alomone labs), 10% fetal bovine serum and 5ng/mL of aphidicolin (A.G. Scientific, San Diego, CA) for 5–7 days. For retrograde transport analysis SCG neurons were cultured in polylysine (100 μg/mL)—laminin (10 μg/mL) using microfluidic devices AXIS Axon Isolation Devices, 450um from Millipore (Billerica, MA)

### Cell culture and HEK-293T transfections

HEK-293T were cultured in MEM, 10% FBS and antibiotics. HEK-293T were transfected with Lipofectamine 2000 (Life Technologies, CA, USA) according to the manufacturer´s instructions, and 6 h after transfection the cells were detached to prepare cell aggregates using the “hanging drop” method [64].

### Treatments of SCG neurons

Dissociated neurons were grown during 5 to 7 days *in vitro* (DIV) and starved of NGF during 16 h when indicated. NGF and vCKBP were mixed in DMEM prior stimulation. To calculate NGF molarity we considered NGF as a dimer (26kDa). The concentrations used were 0.5 nM NGF with 100 nM vCKBP for signaling experiments; 1 nM NGF with 200 nM vCKBP for TrkA-p75 interaction analysis and TrkA internalization assays; and 5 nM NGF with 1 μM vCKBPs were applied in the distal axon compartment for retrograde transport analysis.

### Quantitative colocalization

Pearson´s coefficient (PC) is a standard statistical analysis designed to measure the strength of a linear relationship between two variables, in this case fluorescent intensities from two images. PC generates a range of values from 1, a perfect positive correlation, to −1, a perfect but inverse correlation, with 0 representing a random distribution. The intensity correlation analysis (ICA) [66] method is based on the principle that if two proteins are part of the same complex then their staining intensities should vary in synchrony, whereas if they form part of different complexes or structures they will exhibit asynchronous staining. Intensity correlation quotient (ICQ) was used to provide an overall index of whether the staining intensities were associated in a random, a dependent or a segregated manner. Given that the outlining of regions in which two probes may distribute is required to obtain accurate measurements of colocalization, we drew regions of interest (ROI) corresponding to the entire plasma membrane of the neuron, where the colocalization analysis was carried out. A minimun of three different sections were quantified in the assays shown. ICA analysis was perfomed using Image J 1.43 software.

### *In vivo* infection and transfection of glabrous skin

Mice were anesthetized with a mixture of ketamine xylazine prior to infection. A region located between proximal pads and heel of ventral hindpaw was exfoliated by rubbing the skin 30 times with an exfoliation sponge (3 M, Heavy Duty), followed by tape stripping. This procedure results generally in transient and mild erythema but not in hemorrhage and scar formation [67]. Following exfoliation 10 μL of PBS, HSV-1 or HSV-2 containing 1x10^4^ plaque forming units were applied on the footpad. To transfect mouse hindpaw skin, a similar exfoliation procedure was used. The DNA was transfected using In-vivo Jet Pei (Polyplus Transfection, Illkirch, France) [67].

### FNE staining

Mice were euthanatized 48 h after *in vivo* transfection or infection. Transfected or infected hindpaw skin was immediately removed by using a 3 mm biopsy punch and fixed in Zamboni´s fixative for 6 h. Then biopsies were washed, cryoprotected in 20% sucrose for 24 h, embedded in OCT and sectioned. Staining of FNE was performed by adapting previously established protocols [25,68]. 40 μm free floating sections were washed in PBS with 0.3% Triton X-100 (PBS+TX), blocked for 30 min in 10% horse serum in PBS+TX. Antibodies used for FNE staining were anti-protein gene product (PGP) 9.5 rabbit antibody (Cedarlane-Ultraclone, Ontario, Canada) and anti-CGRP rabbit antibody. Anti V5-tag mouse antibody to detect viral proteins containing a V5 tag was from Sigma. Secondary antibodies used for immunostaining were from Sigma. Confocal analysis was performed with LSM 510 Confocal Laser Scanning Microscope from Carl Zeiss. Analysis and treatment of images was performed using LSM Image Browser, Fiji and Adobe Photoshop.

## Supporting Information

S1 Text It includes information regarding the cells and viruses used in this report and the protocols employed to generate recombinant proteins, to perform crosslinking experiments, western blots, immunofluorescence and immunostaining. It also includes a list of publications related to these materials and methods.
(DOC)

S1 Table It shows the results of the SPR experiments performed with SgG1, SgG2 and M3 and neurotrophic factors. (DOC)

S1 Fig Interaction between vCKBPs and NGF. **(A)** Sensorgrams showing the interaction of Sg1 and M3 with increasing concentrations of NGF. The concentrations of NGF are indicated at the right side of each the sensorgram. **(B)** Sensorgrams showing the interaction of SgG1 and M3 with NGF and the lack of interaction with TNF-α, IFN-α or IL-1. All analytes were injected at a 100 nM concentration. **(C)** Sensorgrams depicting the interaction between increasing concentrations of SgG1 with NGF coupled on a sensor chip. Abbreviations: Diff. Resp., Differential response; R.U., response units; s, seconds. **(D-F)** Crosslinking assays showing the interaction of 1 nM [^125^I]-rNGF with SgG1, SgG2 and M3. **(E, F)** Crosslinking assay between 1 nM [^125^I]-rNGF and SgG2 **(E)** or M3 **(F)** in the presence of increasing concentrations of unlabeled NGF (0, 80, 160 and 320 nM in **E** and 0, 160, 320 and 640 nM in **F**). Molecular masses are indicated in kDa. SgG-NGF complexes are indicated with arrows and non-specific signals are marked with asterisks.
(TIF)

S2 Fig SgG2 disrupts NGF-dependent TrkA-p75NTR interaction. **(A)** Mouse SCG dissociated neurons were grown during 5 DIV. Neurons were deprived of NGF for 16 h and stimulated with NGF, vCKBPs or both during 2 min at 37°C. Following stimulation, cells were PFA-fixed and TrkA-p75NTR interaction at the plasma membrane was analyzed by immunofluorescence without permeabilization. Confocal microscopy images correspond to one representative cell from each condition. A region of the plasma membrane of each neuron is shown in the zoom image. The +ves image displays pseudocolored pixels from the areas within the plasma membrane in which both TrkA and p75NTR pixel value exceed the mean. Scale bar represents 10 μm. **(B)** Pearson´s coefficient (PC) and intensity correlation quotient (ICQ) were calculated for TrkA and p75NTR colocalization. Bar plots show mean±SEM for *n* = 20 cells from two independent assays. Two-tailed unpaired T-test, *P<0.05; **P<0.001. **(C)** Mouse SCG dissociated neurons were grown during 5 DIV. Neurons were deprived of NGF for 16 h and were stimulated with NGF alone or NGF plus SgG2 during 5 min. TrkA-p75NTR interaction was analyzed by TrkA immunoprecipitation followed by western blot to detect p75NTR. The experiment shown is representative of three independent assays.
(TIFF)

S3 Fig Preincubation of neurons with SgG2 it is not sufficient to induce increased TrkA signaling. Mouse SCG dissociated neurons were grown during 5 days *in vitro* (DIV). Neurons were deprived of NGF for 16 h, preincubated with HEPES or 100nM SgG2 for 10 min and then stimulated with 0.5nM NGF and HEPES or 0.5nM NGF and 100nM SgG2 for 15 min. **(A)** The phosphorylation levels of TrkA and ERK were analyzed by Western blot using specific antibodies. **(B)** Graph showing statistical analysis for TrkA phosphorylation (*n* = 6). **(C)** Graph showing statistical analysis for ERK phosphorylation (*n* = 6). **P<0.001; ***P<0.0001; ns: not significant.
(TIFF)
