# Supplementary material for: Secreted Herpes Simplex Virus-2 Glycoprotein G Modifies NGF-TrkA Signaling to Attract Free Nerve Endings to the Site of Infection
Source: PLoS Pathog. 2015 Jan 22;11(1):e1004571. doi: 10.1371/journal.ppat.1004571 (PMC4303327; doi:10.1371/journal.ppat.1004571)
Supplement: S1 Text — It also includes a list of publications related to these materials and methods. (DOC) [file ppat.1004571.s001.doc]

**Supporting Materials and Methods:**

**Cells and viruses:**

Hi-5 insect cells used to generate recombinant SgG1, SgG2 and M3 were routinely maintained at 28^o^C in TC-100 medium and grown in Express Five SFM (Life Technologies, CA, USA) when protein expression by baculovirus infection was required. HEK-293T cells used to express V5-tagged proteins were cultured at 37^o^C in DMEM supplemented with 10% fetal bovine serum. HSV-1 wild type strain SC-16 and wild type HSV-2 strain 333 were a gift from Dr Helena Browne (Cambridge University, UK).

**Generation of recombinant proteins:**

The generation and purification of soluble recombinant proteins (SgG1, SgG2 and M3) expressed with the baculovirus system has been previously described . To express HSV-2 gD we amplified the gD ectodomain using primers HSV-2 gD-1 (5’-ATTCCATGG ATCGATTTCGCGGGAAGAACCTTCCGGTTTTGGACCAGC-3’) and HSV-2 gD-2 (5’-ATTGCATGCTTATGGTGCGGCGCGACGTCCTGGATCGACGGGA TGTGCC-3’), containing *Nco*I and *Sph*I, respectively, from HSV-2 strain 333 DNA. The PCR product was cloned into pFastBacMel and the generation and purification of soluble gD was performed as before for SgG . Briefly, the genes encoding SgG1, SgG2, and the ectodomain of gD were cloned in the pFastBacMel vector (modified from Invitrogen’s pFastBac vector). M3 was cloned into the pBac-1 vector (Novagen Corp.) Transfection of these vectors into insect cells generated the respective recombinant baculoviruses expressing Histidine-tagged SgG1, SgG2, gD or M3, allowing protein purification from the supernatant by affinity chromatrography 72 hours post infection . The following procedure was used to express recombinant proteins in mammalian cells. SPV5-gG1FL is a promoter-free plasmid containing gG2 signal peptide followed by the V5-tag fused to the full-length gG1 protein generated by GeneArt (Life Technologies, USA). The SgG2- and M3-coding sequences were amplified from the plasmids pFastBacMel-SgG2 and pBACM3 , respectively. The amplified fragments were cloned into SPV5-gG1FL replacing the gG1FL sequence by that of SgG2 or M3 resulting in the SPV5-SgG2 and SPV5-M3 constructs, respectively. The fragments SPV5-SgG2 and SPV5-M3 were extracted using *Nhe*I and *Kpn*I and cloned into the expression plasmid pcDNA3.1-Zeo, producing the final plasmids pcDNA3.1-SPV5-SgG2 and pcDNA3.1-SPV5-M3. The constructs were sequenced to discard the presence of mutations.

**Cross-linking experiments:**

Cross-linking experiments were performed as previously described . Purified recombinant SgG1, SgG2 and M3 were incubated with 0.2 μCi of rat [^125^I]-NGFβ (Perkin Elmer) during 30 min. The binding of [^125^I]-NGF to the recombinant proteins was competed with increasing concentrations of unlabeled NGF. The crosslinker BS^3^ was added after 30 min to the mixture and incubated for further 30 min. The complex was resolved in acrylamide gels by SDS-PAGE electrophoresis, fixed with 20% methanol and 10% acetic acid, dried, and exposed to a Kodak film.

**Western blot, immunoprecipitation and immunostaining:**

TuJ1 sc-58888 antibody was from Santa Cruz (CA). Anti-V5 antibody was from Sigma (St. Louis, MO). p-TrkA Tyr490, p-ERK, p-AKT and p-cofilin antibodies were obtained from Cell Signaling (Danvers, MA). Extracellular TrkA AF1056 antibody was purchased from R&D Systems (Minneapolis, MN). Anti-p75 ab38335 used was from Abcam. Pan-Trk antibody was from Santa Cruz. SgG2 was detected with the monoclonal antibody 4a5a2 . For immunoprecipitation 10 μg of SCG neurons extract was incubated overnight with anti-TrkA antibody. Then, the mix was incubated with protein G-coupled agarose beads (GE Healthcare Waukesha, WI) and washed three times with lysis buffer (1% NP40, 50mM Tris pH 7.5, 150mM NaCl and protease and phosphatase inhibitors) prior to analysis by SDS-PAGE and western blotting. For immunofluorescence analysis, neurons were fixed with 4% PFA for 10 min and permeabilized only when indicated. Cholera toxin ß subunit-FITC used to detect GM1 rafts was from Sigma. GM3 polyclonal antiserum was kindly provided by Dr. Illa, Santa Creu I Sant Pau Hospital . Phalloidin-TRITC was purchased to Sigma. Phalloidin Alexa 488, To-Pro-3 and secondary antibodies used were from Life Technologies (Life Technologies, CA, USA). Confocal analyses were performed with LSM 510 Confocal Laser Scanning Microscope from Carl Zeiss (Göttingen, Germany). Analysis and treatment of images were performed using LSM Image Browser, Fiji and Adobe Photoshop.

**Supporting References:**

1. Viejo-Borbolla A, Martinez-Martin N, Nel HJ, Rueda P, Martin R, et al. (2012) Enhancement of chemokine function as an immunomodulatory strategy employed by human herpesviruses. PLoS Pathog 8: e1002497.

2. Parry CM, Simas JP, Smith VP, Stewart CA, Minson AC, et al. (2000) A broad spectrum secreted chemokine binding protein encoded by a herpesvirus. J Exp Med 191: 573-578.

3. Alcami A, Symons JA, Collins PD, Williams TJ, Smith GL (1998) Blockade of chemokine activity by a soluble chemokine binding protein from vaccinia virus. J Immunol 160: 624-633.

4. Shaw G, Morse S, Ararat M, Graham FL (2002) Preferential transformation of human neuronal cells by human adenoviruses and the origin of HEK 293 cells. FASEB J 16: 869-871.

5. Gomez-Mouton C, Abad JL, Mira E, Lacalle RA, Gallardo E, et al. (2001) Segregation of leading-edge and uropod components into specific lipid rafts during T cell polarization. Proc Natl Acad Sci U S A 98: 9642-9647.
