# Supplementary material for: Secreted Herpes Simplex Virus-2 Glycoprotein G Modifies NGF-TrkA Signaling to Attract Free Nerve Endings to the Site of Infection
Source: PLoS Pathog. 2015 Jan 22;11(1):e1004571. doi: 10.1371/journal.ppat.1004571 (PMC4303327; doi:10.1371/journal.ppat.1004571)
Supplement: S1 Table — (DOC) [file ppat.1004571.s002.doc]

| **SgG1** | **Binding** | ***K*_D_ (M)** | ***k_a_*(1/Ms)** | ***k_d_*(1/s)** |
| --- | --- | --- | --- | --- |
| **mbNGF** | Yes | 2.57 x 10^-9^ | 2.83 x 10^5^ | 7.29 x 10^-4^ |
| **hNT-3** | Yes | 3.71 x 10^-8^ | 1.26 x 10^6^ | 4.56 x 10^-2^ |
| **hArtemin** | Yes | 8.33 x 10^-9^ | 5.68 x 10^4^ | 4.73 x 10^-4^ |
| **mGDNF** | Yes | N.C. | N.C. | N.C. |
| **IFN-α** | No | N.A. | N.A. | N.A. |
| **TNF-α** | No | N.A. | N.A. | N.A. |
| **IL-1** | No | N.A. | N.A. | N.A. |
|  |  |  |  |  |
| **SgG2** | **Binding** | ***K*_D_ (M)** | ***k_a_*(1/Ms)** | ***k_d_*(1/s)** |
| **hNT-3** | Yes | 4.15 x 10^-8^ | 4.66 x 10^4^ | 1.94 x 10^-3^ |
| **hArtemin** | Yes | 5.739 x 10^-9^ | 2.3 x 10^6^ | 1.3 x 10^-2^ |
| **mGDNF** | Yes | N.C. | N.C. | N.C. |
| **IFN-α** | No | N.A. | N.A. | N.A. |
| **TNF-α** | No | N.A. | N.A. | N.A. |
| **IL- 1** | No | N.A. | N.A. | N.A. |
|  |  |  |  |  |
| **M3** | **Binding** | ***K*_D_ (M)** | ***k_a_*(1/Ms)** | ***k_d_*(1/s)** |
| **mbNGF** | Yes | 1.54 x 10^-9^ | 2.54 x 10^3^ | 3.97 x 10^-5^ |
| **hNT-3** | Yes | N.C. | N.C. | N.C. |
| **hArtemin** | No | N.A. | N.A. | N.A. |
| **mGDNF** | Yes | N.C. | N.C. | N.C. |
| **IFN-α** | No | N.A. | N.A. | N.A. |
| **TNF-α** | No | N.A. | N.A. | N.A. |

**Table S1:** Interaction of SgG1, SgG2 and M3 with neurotrophic factors. The affinities of some of the interactions are shown. N.A: not applicable; N.C: not calculated.
