# Supplementary figures and images for: Secreted Herpes Simplex Virus-2 Glycoprotein G Modifies NGF-TrkA Signaling to Attract Free Nerve Endings to the Site of Infection
Source: PLoS Pathog. 2015 Jan 22;11(1):e1004571. doi: 10.1371/journal.ppat.1004571 (PMC4303327; doi:10.1371/journal.ppat.1004571)

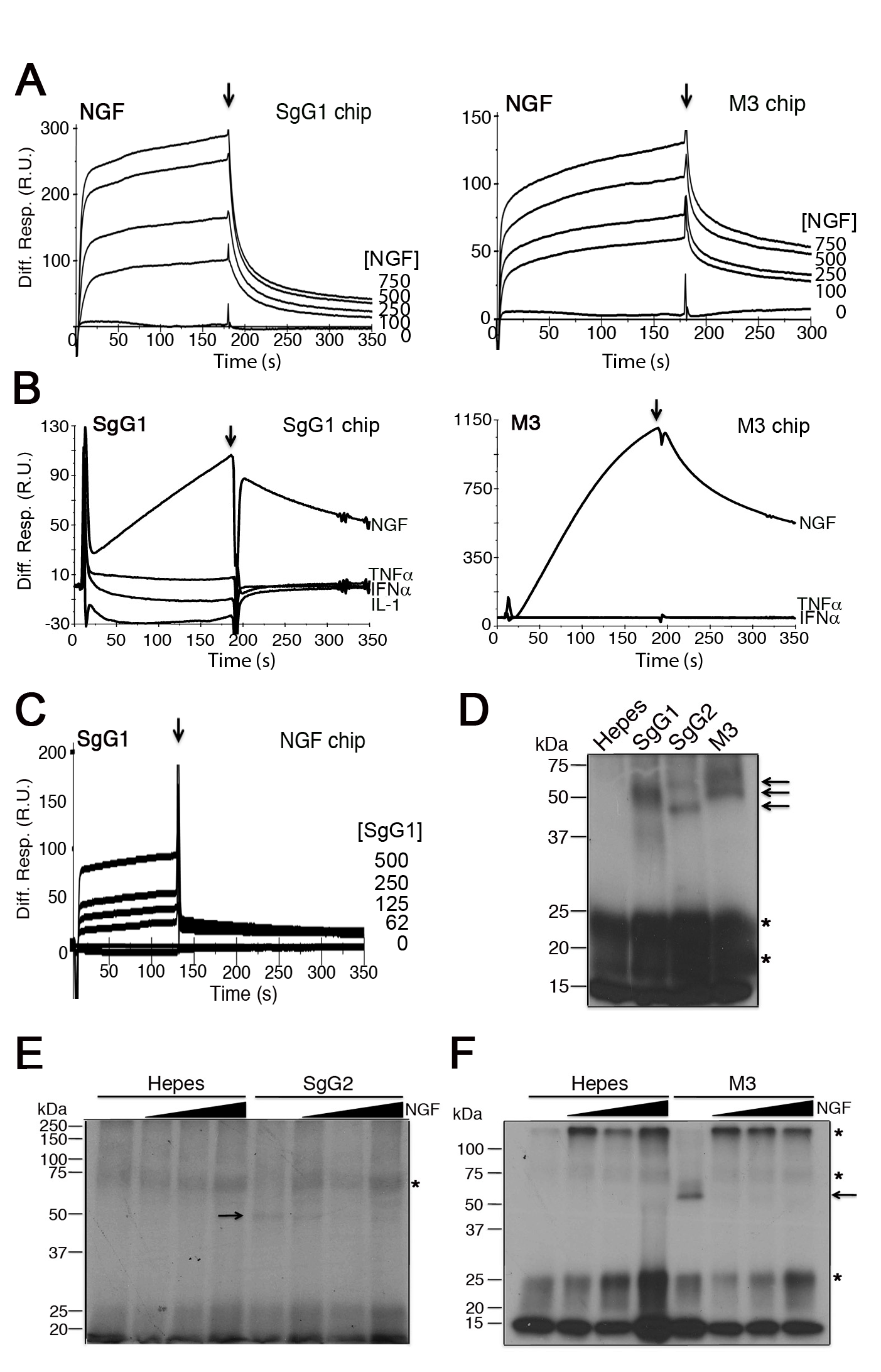

Supplement: S1 Fig — (A) Sensorgrams showing the interaction of Sg1 and M3 with increasing concentrations of NGF. The concentrations of NGF are indicated at the right side of each the sensorgram. (B) Sensorgrams showing the interaction of SgG1 and M3 with NGF and the lack of interaction with TNF-α, IFN-α or IL-1. All analytes were injected at a 100 nM concentration. (C) Sensorgrams depicting the interaction between increasing concentrations of SgG1 with NGF coupled on a sensor chip. Abbreviations: Diff. Resp., Differential response; R.U., response units; s, seconds. (D-F) Crosslinking assays showing the interaction of 1 nM [125I]-rNGF with SgG1, SgG2 and M3. (E, F) Crosslinking assay between 1 nM [125I]-rNGF and SgG2 (E) or M3 (F) in the presence of increasing concentrations of unlabeled NGF (0, 80, 160 and 320 nM in E and 0, 160, 320 and 640 nM in F). Molecular masses are indicated in kDa. SgG-NGF complexes are indicated with arrows and non-specific signals are marked with asterisks. (TIF) [file ppat.1004571.s003.tif]

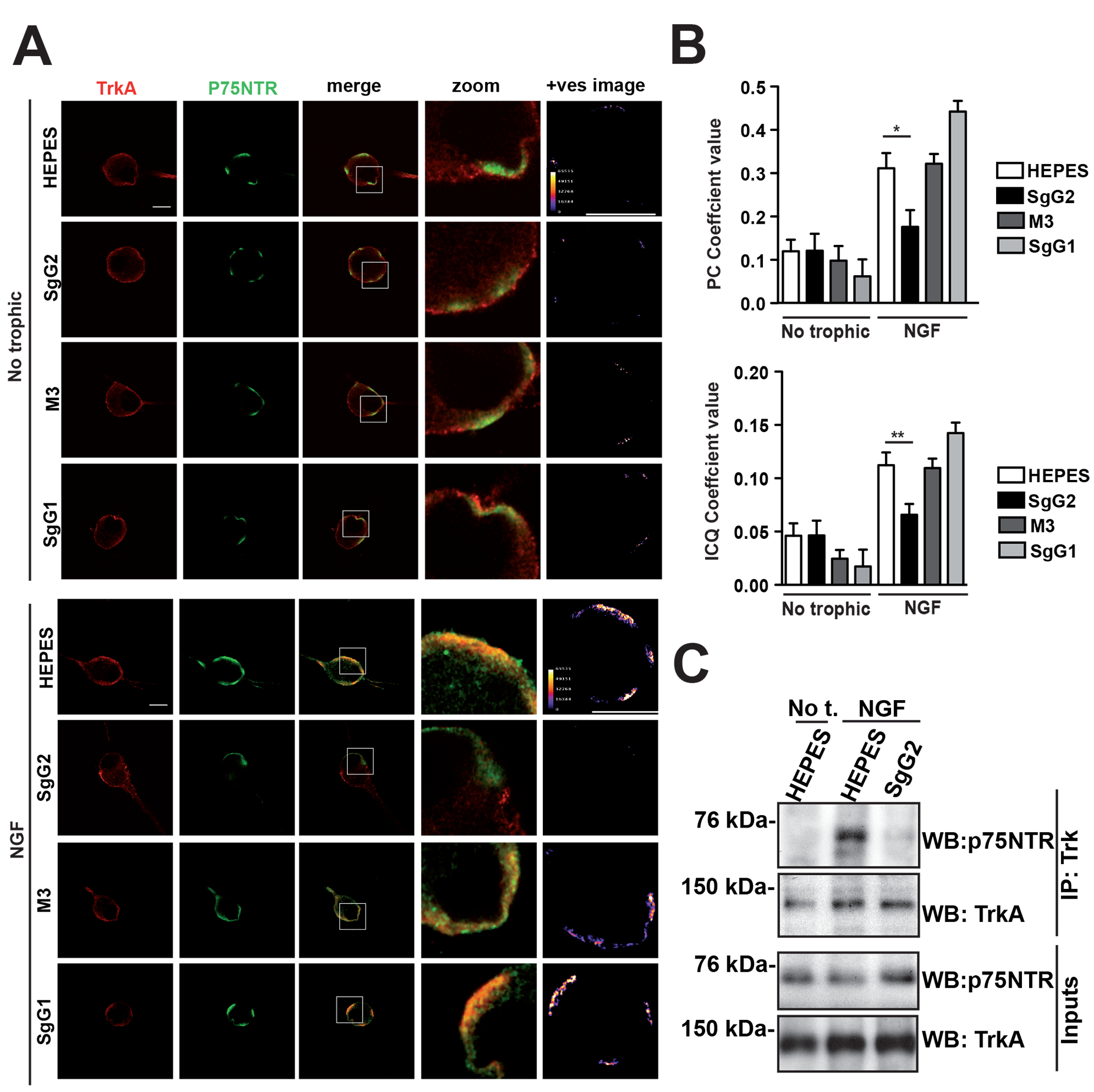

Supplement: S2 Fig — (A) Mouse SCG dissociated neurons were grown during 5 DIV. Neurons were deprived of NGF for 16 h and stimulated with NGF, vCKBPs or both during 2 min at 37°C. Following stimulation, cells were PFA-fixed and TrkA-p75NTR interaction at the plasma membrane was analyzed by immunofluorescence without permeabilization. Confocal microscopy images correspond to one representative cell from each condition. A region of the plasma membrane of each neuron is shown in the zoom image. The +ves image displays pseudocolored pixels from the areas within the plasma membrane in which both TrkA and p75NTR pixel value exceed the mean. Scale bar represents 10 μm. (B) Pearson´s coefficient (PC) and intensity correlation quotient (ICQ) were calculated for TrkA and p75NTR colocalization. Bar plots show mean±SEM for n = 20 cells from two independent assays. Two-tailed unpaired T-test, *P<0.05; **P<0.001. (C) Mouse SCG dissociated neurons were grown during 5 DIV. Neurons were deprived of NGF for 16 h and were stimulated with NGF alone or NGF plus SgG2 during 5 min. TrkA-p75NTR interaction was analyzed by TrkA immunoprecipitation followed by western blot to detect p75NTR. The experiment shown is representative of three independent assays. (TIFF) [file ppat.1004571.s004.tiff]

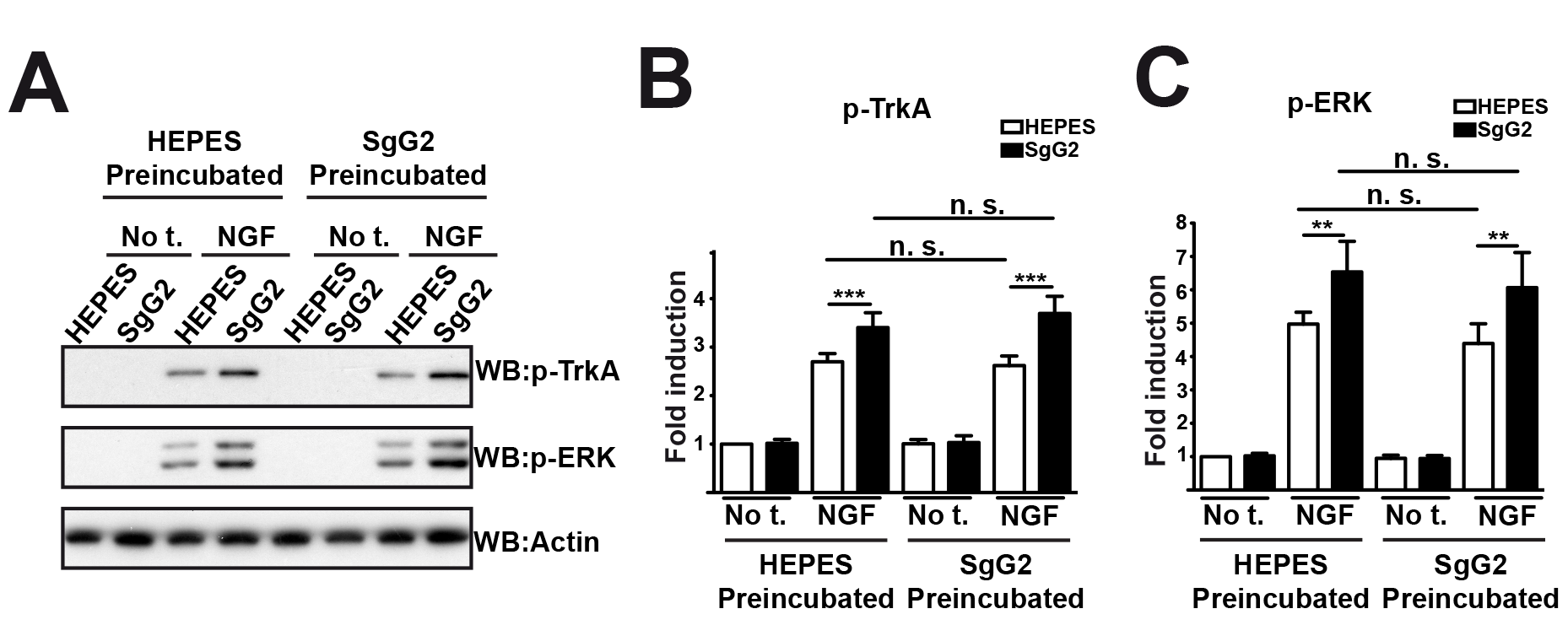

Supplement: S3 Fig — Mouse SCG dissociated neurons were grown during 5 days in vitro (DIV). Neurons were deprived of NGF for 16 h, preincubated with HEPES or 100nM SgG2 for 10 min and then stimulated with 0.5nM NGF and HEPES or 0.5nM NGF and 100nM SgG2 for 15 min. (A) The phosphorylation levels of TrkA and ERK were analyzed by Western blot using specific antibodies. (B) Graph showing statistical analysis for TrkA phosphorylation (n = 6). (C) Graph showing statistical analysis for ERK phosphorylation (n = 6). **P<0.001; ***P<0.0001; ns: not significant. (TIFF) [file ppat.1004571.s005.tiff]
